# Oxytocin: A Multi-Functional Biomolecule with Potential Actions in Dysfunctional Conditions; From Animal Studies and Beyond

**DOI:** 10.3390/biom12111603

**Published:** 2022-10-31

**Authors:** Anastasia S. Tsingotjidou

**Affiliations:** Laboratory of Anatomy, Histology and Embryology, School of Veterinary Medicine, Faculty of Health Sciences, Aristotle University of Thessaloniki, 54 124 Thessaloniki, Greece; astsing@vet.auth.gr; Tel.: +30-2310999941

**Keywords:** oxytocin, oxytocin secretion, cardiovascular diseases, SARS-CoV-2, autism

## Abstract

Oxytocin is a hormone secreted from definite neuroendocrine neurons located in specific nuclei in the hypothalamus (mainly from paraventricular and supraoptic nuclei), and its main known function is the contraction of uterine and/or mammary gland cells responsible for parturition and breastfeeding. Among the actions of the peripherally secreted oxytocin is the prevention of different degenerative disorders. These actions have been proven in cell culture and in animal models or have been tested in humans based on hypotheses from previous studies. This review presents the knowledge gained from the previous studies, displays the results from oxytocin intervention and/or treatment and proposes that the well described actions of oxytocin might be connected to other numerous, diverse actions of the biomolecule.

## 1. Introduction

**Figure 1:**
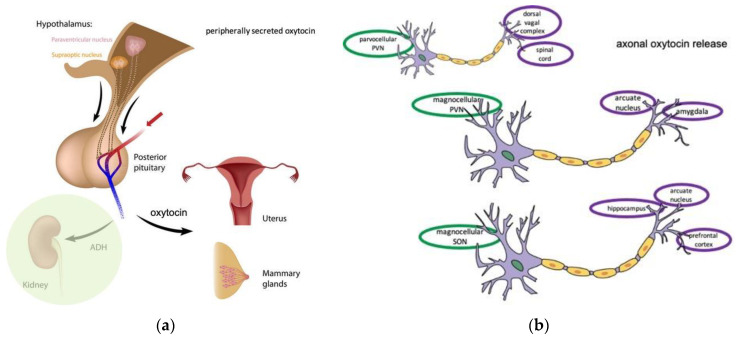
Diagram showing the peripherally secreted oxytocin (**a**) and the axonal oxytocin release (**b**).

Oxytocin is a nonapeptide, with peripheral and central actions. Sir Henry Dale, in 1906, discovered this molecule which being secreted by a human posterior pituitary gland extract caused contractions in a pregnant cat’s uterus; hence he named it oxytocin, a Greek word meaning fast (quick) parturition [1]. It was later reported by Ernst Scharrer in 1928 in a study on fish neuroendocrinology [2], and was the first neuropeptide to be characterized by Du Vigneaud in the 1950s [3], which was later evaluated as an evolutionary tracer by Acher [4]. Its name is indicative of its involvement in parturition, as well as the descent of milk during breastfeeding, in humans [5] and animals [6,7]. It is produced by the neuroendocrine cells of the hypothalamus, in the paraventricular (PVN; mainly from its magnocellular cells) and the supraoptic nucleus (SON) [8,9,10]. The derivatives of the two nuclei are transported through the neurohypophysial bundle to the posterior lobe of the pituitary gland [11,12,13]. The peripherally secreted oxytocin acts into the uterine smooth muscle cells [14] and into the muscle-like cells (myoepithelial cells) into the breast [15,16] (Figure 1a). Along with the magnocellular PVN cells producing oxytocin, parvocellular and the accessory nuclei cells from the PVN also produce oxytocin [17]. Another hormone, the antidiuretic hormone (ADH; shaded circle in Figure 1a) [18], is produced by the same nuclei in the PVN as oxytocin [19] and shares similar, but also different, actions with it. 

Centrally, oxytocin acts as a neurotransmitter or neuromodulator [20] through axonal and/or dendritic release. Other than this secretion of oxytocin, the presence of oxytocin receptors is indicative of its diverse functions. In addition to the two major areas of production, oxytocin is also synthesized in some species in additional sites, including the bed nucleus of the stria terminalis (BNST), the medial preoptic area (MPOA), the lateral hypothalamus (LH) and the medial amygdala (MeA) [21,22]. Axonal projections from the same areas of PVN and SON project in various parts of the brain (Figure 1b): amygdala, arcuate nucleus (from the magnocellular parts of PVN = mPVN), the dorsal vagal complex (from the parvocellular part of PVN = pPVN) and to the prefrontal cortex, hippocampus and arcuate nucleus (from the magnocellular parts of SON = mSON) [23]. On the other side, dendritic projections from the parvocellular part of PVN project at least, into amydgala [24], along with projections from SON. Additionally, there has been verified paracrine modulation of parvocellular nucleus neuronal activity by somato-dendritic secretion from magnocellular neurosecretory cells [25]. A comprehensive list of brain areas that have been identified to express oxytocin receptors in the rat and mouse brain can be found into the important review of Jurek and Neumann [26]. In another review by Grinevich et al., the spatial distribution of oxytocin receptors and oxytocin axons in the rat brain is depicted [27].

## 2. Secretion of Oxytocin and Its Consecutive Functions

Oxytocin (**OXT**) is a very abundant neuropeptide, exerting a wide spectrum of central and peripheral effects as a neurohormone, neurotransmitter or neuromodulator.

### 2.1. From the Brain, in the Periphery

As we have briefly discussed in the previous paragraph, oxytocin neuroendocrine neurons project via their axons into the posterior pituitary gland [28]. Through blood circulation, the secreted oxytocin reaches the uterine smooth muscles and their subsequent contraction facilitate parturition and postpartum recovery of uterus [29,30]. The uterus is also influenced by parasympathetic and sensory processing outflow through oxytocin-immunoreactive neurons of the PVN projecting to the lumbosacral spinal cord [31].

Oxytocin is also responsible for the contraction of the myoepithelial cells surrounding the mammary alveoli which lead to the milk let-down. The pattern of oxytocin release exhibits species specific characteristics and can vary significantly [32]. In humans, this milk ejection reflex appears 30–60 s after the infant has begun suckling and is viable during the whole time a mother breastfeeds her child [13]. The reflex is centrally activated even before the infant begins to suckle, triggered by other external stimuli, e.g., the infant crying which causes a dendritic release of central oxytocin [33]. This external trigger mechanism of the milk ejection reflex has been shown to be an important factor for successful, long-lasting breastfeeding [13]. In sheep, the neuroanatomical basis of the milk ejection reflex has been elucidated [6,34,35]. Electrophysiological studies also exist to describe the milk-ejection reflex in the sheep [36] where oxytocin is released only once or twice during each suckling session. The fact that oxytocin neurons are unaffected by the suckling efforts between successive milk ejections suggests the existence of a mechanism which somehow interrupts the input from the nipples before it reaches its hypothalamic destination [37]. In rodents, where animals can be genetically engineered, milk let-down in oxytocin knockout mice is unavailable for the pups [38], while in knockout mice that were lacking the oxytocin receptors, although the parturition process was normal, lactation and the subsequent maternal nurturing were elusive [39]. It has long been found that suckling stimulation creates neural signals not only from mammary gland, but from other organs as well, including gastrointestinal tract and olfactory bulbs, that reach the hypothalamus [40] and a synchronization center in the ventroposterior hypothalamic area [41]. The latter activates simultaneously the oxytocinergic neurons in the PVN and SON [42], conducting a massive discharge of oxytocin and the milk ejection from the mammary gland [43]. Other physiological processes necessitating pulsatile oxytocin secretion, such as orgasm and ejaculation, as well as tonic uterine contraction during parturition, are all under the control of the same synchronization center. Thus, studying the milk-ejection reflex remains the best model for clarifying the regulation of OXT secretion [44].

Systemic secretion of oxytocin also affects gut motility and (in rats) sodium excretion from the kidneys. Pancreas and adipocytes also express oxytocin receptors and may be targets for circulating oxytocin [45]. Oxytocin exerts its function via its receptor, which is a typical class of I G protein-coupled receptor [13,46]. The existence of OXT receptors (OXTRs) are presented in the publication of Jurek and Neumann [26] where, among others, these molecules have been identified mostly in rodents: in neonate mice, in their anogenital region, adrenals, eye, oronasal cavity [47] and whisker pads; in mice, in the medulla of adrenal glands, taste buds, osteoblasts and osteoclasts, adipocytes and retina; in rats, in cardiomyocytes, testes, penis, uterus, enteric neurons and enterocytes, nociceptive dorsal root ganglion neurons, macula densa cells of the renal cortex and adipocytes. In humans, oxytocin receptors have been identified in cardiomyocytes, dermal fibroblasts and keratinocytes, osteoblasts and osteoclasts. Lastly, there is one publication stating the presence of oxytocin receptors in the medulla of bovine adrenal glands.

### 2.2. From the Brain, in the Brain

Among species, the distribution of oxytocinergic neurons in the brain is relatively consistent but their projections is a species-specific characteristic, while the distribution of OXTRs is very variable [45]. The considerable species variation in OXTR expression reflects differences in the oxytocin receptor gene [48], which is associated with differences in reproductive behavior. Other than the two major areas of production, oxytocin is also synthesized, in some species in additional sites, as mentioned previously [21,22]. Parvocellular PVN (pPVN) axonal discharge, however, is also communicated to different brain areas: the amygdala, nucleus accumbens (nAcc), dorsal vagal complex in the caudal brainstem and to the spinal cord [45,48,49]. Neurons in the previously mentioned areas contain OXTRs and they are responsible for integrating energy balance. mPVN and SON axons project in the arcuate nucleus [45], while in rats, oxytocin neurons project to all main regions of the forebrain [27]. Other areas of axonal projection of oxytocinergic neurons are limbic sites, such as the hippocampus, striatum, and mid- and hindbrain nuclei, including the locus coeruleus and nucleus of the tractus solitarius [20]. Oxytocin released within the brain itself is thought to regulate behavior by acting as a neurotransmitter. Its central role in the regulation of affiliative behavior and social bonding in animals is well known [20].

On the other hand, their dendrites secrete oxytocin (as a neuromodulator) into different brain areas. Sites such as the ventromedial nucleus (VMN) and amygdala contain very few oxytocin fibers, but are likely to be accessed by extrasynaptic oxytocin release, including oxytocin secreted from the dendrites of magnocellular neurons in the SON and PVN [45]. In rats, the olfactory bulb, ventral pallidum, medial preoptic area and VMN are realizing dendritically oxytocin most likely due to their location at the vicinity of PVN and SON, especially in the last three of these regions. The olfactory bulb seems likely to receive oxytocin via the cerebrospinal fluid (CSF). Of these sites, the VMN appears to be the most evolutionarily conserved region of receptor expression. Oxytocin receptor binding is prominent in the VMN in mice, rats, guinea pigs, prairie and montane voles and in rhesus monkeys [50] and humans [51]. 

One of the numerous sites of identified oxytocin receptors is the anterior pituitary gland. More specifically, the release of oxytocin into the vicinity of the long portal vessels connecting the hypothalamus with the anterior pituitary gland and the presence of short portal vessels connecting the posterior lobe to the anterior pituitary influences the release of one or several adenohypophyseal hormones [52]. The participatory role for oxytocin in the physiologic regulation of at least two hormones, prolactin and adrenocorticotropin, has been evidenced. Oxytocin may indirectly control gonadotroph function as well [53]. Oxytocin receptors have also been identified in other tissues, including the kidney, heart, thymus, pancreas and adipocytes [46]. Hence, OXT is also implicated in cardiovascular regulation, body temperature regulation, feeding and gastric distension [54].

### 2.3. From Other Cells and Organs

Oxytocin is produced locally in specialized cells of the uterus, amnion, chorion and decidua, where it acts as a paracrine signal to influence the behavior of neighboring cells [30,55]. It is secreted also in the corpus luteum, testis and heart [46], as well as from the gastrointestinal tract, epididymis and prostate, thymus and adrenal medulla [56]. It is a potent stimulator of spontaneous erections in rats and is involved in ejaculation [46]. Oxytocin and its receptors are found in the thymus [57] and in macrophages [58] as well. The fact that OXTRs are present in macrophages and that their genes include response elements for interleukins and acute-phase reactants [13] indicates the involvement of oxytocin in modulating inflammatory and immune processes. In addition, oxytocin showed anti-inflammatory properties by alleviating paw edema induced by carrageenan [59]. Exogenous oxytocin administration also reduces tissue damage in a variety of animal models of injury [60,61]. Moreover, the co-administration of an OXTR antagonist blocks the protective effects of oxytocin during cardiac ischemia [61] or cerebral ischemia in rats [59,62].

Since OXT producing cells are present in all the above-mentioned tissue (heart, brain and gastrointestinal tract) and extensive expression of OXTRs, therapeutic effects of oxytocin are connected to a number of affiliated diseases since this biomolecule has immunomodulatory, cardioprotective, anti-diabetic and anabolic functions, as well as psycho-social functions [63].

## 3. Use of Oxytocin in Dysfunctional Conditions

Taking into consideration the anti-inflammatory and antioxidant properties of OXT [64,65,66], it is possible for it to have therapeutic merit, even in the dysfunction of nervous system. Oxytocin’s secretion reacting to different physiological stimuli also contributes significantly in response to stress and in reducing proinflammatory responses in diverse milieus, such as neurodegenerative disorders [67]. 

In the present study, we will discuss interventions and potential therapeutic actions of this multi-functional molecule, from the observations we have from *in vitro*, *in* and *ex vivo* and human studies. We present an example from disorders of the nervous system (autism), cardiovascular disease and the obesity/diabetes disorder. Although obesity is linked both to cardiovascular disease and diabetes, we are presenting this disorder along with diabetes. At the end, since Sars-CoV-2 has disarrayed our lives fiercely and its outcome (of COVID-19) is negatively connected with underlying conditions such as diabetes and/or cardiovascular disease, we will give a possible role of oxytocin as therapeutic agent based on the previous scientific knowledge. 

**Figure 2:**
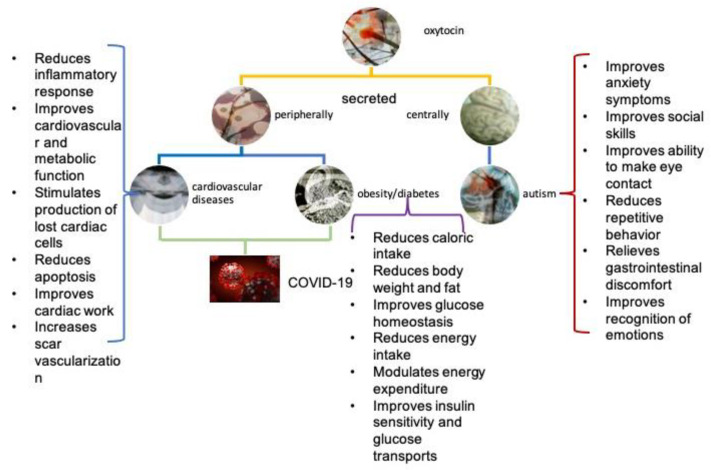
Diagram showing the diseases discussed in this manuscript which might be affected by the use of oxytocin. The key role of oxytocin is mentioned in each situation. The photographs (except the one of COVID-19) are from the video *Tender Morality*, by Stavros Panagiotakis.

The presentation of the diseases discussed in the manuscript is in Figure 2.

### 3.1. In Autism

Autism, or, more correctly, the autism spectrum disorders (ASD), is a neurodevelopmental disorder underlying neurodegenerative causes. According to the WHO, conditions that are characterized by some degree of difficulty with social interaction and communication [68] are specified as ASD. These are a diverse group of diseases with additional characteristics being atypical patterns of activities and behaviors, such as difficulty with transition from one activity to another, a focus on details and unusual reactions to sensations. About 1 in 160 children has an ASD [69]. The classification of autism as a degeneration disorder [70] is driven from the fact that its manifestation holds a few events associated with neurogenerative characterization, such as Purkinje cell loss degeneration combined with microglial activation [71] or gliosis [72].

Oxytocin is involved as a regime for ASD’s treatment. In the following paragraphs, representative published basic research on the field will be presented along with the studies and pharmaceutical interventions in humans.

Among oxytocin’s, and its receptors’, effects in behavior are social memory and recognition, affiliation (sexual, maternal and paternal), aggression (female and male) and other non-social behaviors, such as learning and memory, anxiety and depression [73,74].

From earlier studies [75], the positive relationship between OXT and formation of social bonds in animals, which led to speculate that its abnormalities may be involved in autism, has been shown. Actually, several studies indicate that single nucleotide polymorphisms (SNPs) in the OXT and OXTR genes are linked with ASD [76,77,78,79]. Intravenous infusion of OXT into adults with autism and Asperger’s disorder significantly reduces both number and severity of repetitive behaviors (such as repeating, self-injury and touching) [80] and increases ability to comprehend and remember the affective component of spoken words (happy, indifferent, angry or sad) [81].

However promising the link between oxytocin and autism may be, it is important to remember that many gene systems, in many combinations, contribute to any observable phenotype, and that many systems are currently being explored in relation to autism [82,83,84,85]. Marazziti and Vecchia describe, in a recent review, the possible link between OXT and autism, as well as other neuropsychiatric disorders [86].

Tachibana et al. [87] had shown that the intranasal administration of OXT is safe for use in adolescents with autism and they do suggest this therapy to be safe, promising and worthy of a large-scale, double-blind placebo-controlled study [87], even though their data were too preliminary to draw any definite conclusions about efficacy. Around the same time, Anagnostou et al. [88], in their pilot study, suggest that daily administration of intranasal oxytocin at 0.4 IU/kg/dose in children and adolescents with ASD is safe and has therapeutic potential. They also provide a review on numerous clinical trials that are used in the autism related disorders in humans, and especially in young adults. The first evaluation of the efficacy for a course of oxytocin treatment for youth with ASD was conducted by Guastella et al., although their results did not suggest clinical efficacy. However, they concluded that further research was needed to explore alternative delivery methods, earlier age of intervention, and the influence of caregiver expectation on treatment response [89]. Since then, researchers concluded that subsequent clinical trials of longer-term administration have yielded more conservative and mixed evidence [90] and a systematic review reported adverse events of long-term intranasal oxytocin in treatment of ASD [91]. In another review from Gulliver et al. [92], the authors target neuropsychiatric disorders and their possible treatment with oxytocin, and an updated meta-analysis of randomized controlled trials is published regarding oxytocin therapy for core symptoms in ASD [93]. A cross-disciplinary systematic review on chronic oxytocin administration as a tool for investigation and treatment [94] and a multilevel meta-analysis on intranasal oxytocin in the treatment of autism spectrum disorders are the more recent publications on the issue [95]. Additionally, another recent study of Sikich et al. showed limited therapeutic potential as an intervention for children with ASD [96]. 

The previously mentioned use of OXT in the therapy of autism in humans is based on the results of plentiful animal studies, mainly performed in rodents, and especially in mice. In the most recent review on the animal models used for autism [97], there is detailed information on these in vivo studies, in both genders with genetically engineered interventions, with Ferguson and colleagues being the first to report that OXT-deficient mice display a specific impairment in social recognition memory [98]. Since Munesue et al. reported that the tested two variant *CD38* poloymorphysms may be of interest with regard of the pathophysiology of ASD [99], this molecule has been used in autism animal models [97]. Jin et al. [100] showed the impairment of social recognition memory in CD38 deficient mice. Additionally, the same murine lines presented with different behavioral autism-like manifestations, such as separation-induced pup vocalization [39,101], which is thought to represent linguistic skills in animal models and cognitive flexibility, related to stereotyped behavior, as well as susceptibility to seizures [102]. Interestingly, a recent study showed that heterozygous OXTR mice [103] display some social behavior deficits, suggesting that these behaviors are sensitive to OXTR gene dosage. Numerous genetically engineered mice have contributed to the study of oxytocin link with ASD in animals, with the more recent one, where *Magel2tm1.1Mus*+/+ (WT) and *Magel2 tm1.1Mus*−/− (*Magel2*-KO) mice demonstrated the positive effects of subcutaneous administration of oxytocin in the mutant neonates, restoring hippocampal alterations and social memory at adulthood [104]. *Magel1*-deficient mice develop progressive obesity, and other pro-autism behaviors along with a significant decrease in the production of mature oxytocin in the brain, which was reversed following acute subcutaneous administration of oxytocin leading to rescue of their social memory deficits [105]. *Magel2*-deficient mice also exhibit feeding difficulties as well as deficits in social behavior and learning [106,107]. Similar to *Magel1*-deficient mice, *Magel2*-deficient pups show a significant reduction in the production of mature oxytocin in the PVN, while intermediate forms of the peptide are enhanced [107].

Other than the animal models, where components of the OXT system were genetically targeted, leading to ASD-associated deficits, validated ASD animal models, where impairments in the oxytocin system were detected, are also used. These include studies supporting the role of OXT in mediating the GABA switch during early development and suggest that failure of this switch, due to different factors, leads to the demonstration of ASD symptoms in animal models. Among these are the genetic model of FXS; the Fmr1-KO mouse, and the pharmacologically induced model of ASD; and the prenatally VPA-exposed rats [108]. In this work, the authors propose that failure of the oxytocin-mediated process regulating the GABA switch around birth causes the development of ASD symptoms in several ASD animal models and, thus, may also be involved in ASD etiology. Thus, their results validated the clinical actions of oxytocin [109] and emphasize the importance of investigating how and when developmental sequences are disrupted in animal models of autism. 

Additionally, other ASD animal models, are based on oxytocin administration that was found to alleviate ASD-associated impairments. These are studies where researchers examined the effects of administration of oxytocin on two inbred mouse lines, BALB/cByJ and C58/J with ASD-related behavioral deficits exhibited [110] or on *Grin1*-defiecient mice (which lacks the N-methyl-D-aspartate receptor NR1 subunit) with various therapeutic effects [111]. In *Oprm1*-KO mice (which lack the mu 1 opioid receptor) [112] or in *Stx1a*-KO adult male mice (lacking Syntaxin 1a) [113], different effects on the impairment of ASD symptoms were observed. In the work of Harony-Nicolas et al. where *Shank3*-deficient rat model (leads to synaptic plasticity deficits) was found that intracerebroventricular injection of oxytocin could rescue the synaptic plasticity deficits and ameliorate the impaired behavior [114].

*In vitro* models, as well *ex vivo* studies, for researching oxytocin in autism do not exist. The only *in vitro* model is on a gene for a specific vasopressin receptor, V1aR, in prairie voles, which exhibit monogamous behavior [115].

The studies, *in vitro*, *in* and *ex vivo*, for all the degenerative diseases discussed in this manuscript (autism, cardiovascular diseases, obesity/diabetes and COVID-19) are listed in Table 1.

### 3.2. In Cardiovascular Diseases: (High) Blood Pressure-Heart Rate-Muscle Contraction

Cardiovascular diseases (CVDs) according to the WHO, are responsible for 17.9 million peoples’ deaths each year, which accounts for 32% of all deaths [173]. CVDs are a group of disorders of the heart and blood vessels and include coronary heart disease, cerebrovascular disease, rheumatic heart disease and other conditions. It is also known that more than four out of five CVD deaths are due to heart attacks and strokes, and one third of these deaths occur prematurely in people under 70 years of age. Although new technology diagnostic and invasive tools have contributed vastly to the confrontation of these diseases, there is still space for possible therapeutic strategies in the field. New interventions and strategies for the use of oxytocin in these diseases are discussed in the following paragraphs.

Oxytocin is affiliated to the protection of cardiovascular function [174,175]. More specifically, the heart produces and releases oxytocin which acts on its cardiac receptors to decrease heart rate and force of contraction [176]. Systemic administration of OXT has significant effects on blood pressure, vascular tone and cardiovascular regulation. Oxytocin has been linked to both increase and decrease in blood pressure [176,177,178,179], while systemically administered oxytocin induces a short-lasting increase in blood pressure in rats [180,181]. No effect on vascular tone in vessels of pregnant and nonpregnant rats has been reported [182]. The opposite effect is documented in humans [183,184]. It has been also shown that oxytocinergic neurons are present in various brain areas related to cardiovascular regulation, such as nuclei of the pontine and medullary respiratory centers [185,186,187]. PVN also projects directly to the sites that control cardiorespiratory function, such as intermediolateral cell columns, phrenic motor nuclei in the spinal cord, rostral ventrolateral medulla, and the rostral nuclei in the ventral respiratory column in the brainstem [44]. Additionally, oxytocin receptor transcripts and oxytocin binding sites have been demonstrated in atrial and ventricular sections of the heart [188] and in vascular endothelial cells [171,178]. Moreover, besides the hypothalamus and other parts of the brain, the rat heart has been shown to be a site of oxytocin synthesis and release [177,189]. 

From the existing in vitro studies in cultured human vascular cells, THP-1 monocytes and macrophages, it is shown that oxytocin alleviates vascular oxidative stress and inflammation, two important pathophysiological processes in atherosclerosis and subsequently into cardiovascular disease [116]. Moreover, OXTs are found in monocytes and macrophages, and oxytocin decreases both superoxide production and release of a proinflammatory cytokine from these cells, suggesting a potentially larger role for oxytocin in the attenuation of disease [116]. LPS effect (indicative of cardiovascular disorder) has also been tested in cells lines, with a dramatic decrease following OXT treatment [58]. In newborn and adult rat cardiomyocyte cultures, OXT induced an anti-hypertrophic response [117]. In newborn rat cardiomyocytes, umbilical cord blood-derived mesenchymal stem cells (UCB-MSCs), combined with OXT may contribute to the cardiogenic potential for cardiac repair [118]. In another type of cells, the heart-derived H9c2 cells, OXT treatment in ischemia/reperfusion was more effective in early reperfusion. Activation of the responsible pathways were also revealed in the same study [132,187]. 

Next to the previously mentioned *in vitro* studies with the use of different cell types, including neonatal and adult myocytes, stand the use of numerous animal studies. Peripheral OXT administration in stable group housing conditions ApoE−/− mice indicates an inhibition of atherosclerotic lesion development and adipose tissue inflammation [119]. Infusion of OXT into Sprague Dawley rats improved function of their injured heart by reducing inflammation and apoptosis in infracted myocardium [120]. Recently, the same strain of rats was used in a study where the activation of cardiac mast cells and other inflammatory factors participated in the protective activity of OXT in an ischemia/reperfusion injury model [121]. In an experiment where rats received a trans-ascending aortic constriction (TAC)-induced heart failure, the activation of hypothalamic OXT neurons to elevate parasympathetic tone led to reduced cellular hypertrophy, levels of IL-1b, and fibrosis. Additionally, cardiac contractility parameters were significantly higher in TAC+OXT compared with TAC animals. Heart rate sensitivity, but not contractile sensitivity, to β-adrenergic stimulation was improved in TAC+OXT hearts [122]. In another experiment in rats, it was shown that pre autonomic oxytocin neurons can drive the increase in cardiac sympathetic nerve activity following myocardial infraction and peripheral administration of an OXTR blocker [123]. Japanese white rabbits treated postinfarct with OXT showed reduction in myocardial infract size and improved left ventricular function and remodeling [124]. Chronic peripheral OXT administration in Watanabe Heritable Hyperlipidemic (WHHL) rabbits can inhibit inflammation and atherosclerotic lesion development [125]. In a porcine myocardial infract model, pretreatment of the animals seems to impact the outcome of the infraction [126].

Ondrejcakova et al. [127] demonstrated an attenuation of the infarct size in OXT-treated hearts, indicating a cardioprotective effect of oxytocin. The data suggest that the negative chronotropic action of oxytocin participates in its protective effects on ischemia–reperfusion-induced myocardial injury [127]. Oxytocin has been also shown to exert cardiac postconditioning. The effect was dose dependent in an isolated rat heart model [128]. Intracerebroventricular administration of OXT resulted in a preconditioning effect in ischemic-reperfused rat heart [129], a model which was tested earlier where activation of cardiac OXTRs by stress led to cardioprotection [130]. Studies on an isolated heart are numerous (more details at Jankowski et al., 2020 [175]), including one on a dog heart [131] where it was found that OXT holds inotropic and chronotropic effects which are mediated by cardiac oxytocin neurons.

Human studies on the use of oxytocin in the prevention of cardiovascular diseases are combined with its therapeutic use for the treatment of obesity and/or diabetes, which are presented in the next paragraph.

### 3.3. In Obesity (Food Intake and Satiety)/in Diabetes (Glucose Uptake-Insulin Secretion)

The World Health Organization recognizes obesity, raised BMI (body Mass Index) [190], as a major risk factor for noncommunicable diseases such as: cardiovascular diseases (mainly heart disease and stroke), diabetes, musculoskeletal disorders (especially osteoarthritis) and some cancers (including endometrial, breast, ovarian, prostate, liver, gallbladder, kidney and colon).

Diabetes, a metabolic disorder with major degenerative complications will be discussed in this manuscript in conjunction with obesity since OXT functions share therapeutic effects in both diseases. For this reason, we will present several studies with the use of oxytocin on obesity and diabetes, since these are closely related.

For the detailed involvement of OXT pathways in and outside of the brain in the control of energy balance, see the review of Niu et al. [191]. In brief, this is based on the combination of species-specific OXT expression within other areas than the primary OXT expression by pPVN neurons and the m(PVN and SON); the collaterals from the PVN and SON oxytocin neurons in the brain; and the secretion of OXT from nerve terminals, the reactivity of OXT neurons to molecules, such as the adiposity signal, leptin and/or CCK and NPY, and the fact that oxytocin is expressed in peripheral tissues, such as heart, GI tract, islets of Langerhans of the pancreas and Leydig cells in the testes. 

In 2015, Blevins and Baskin [192] reported that in the list of the funded or to be funded investigations, were almost 400 mentioning OXT and obesity or obesity related conditions. In a very recent paper of Niu et al. [191], this number has been raised in 535 completed, ongoing and pending investigations in humans. It is beyond the scope of this review to present all the human studies using OXT as a therapeutic intervention in obesity. We will try though to discuss the most recent human studies. 

In addition to the concerns about sex-specific results from OXT treatment [193], careful selection of the conditions in which oxytocin treatment should be beneficial for obesity and its comorbidities, and their relevance for human pathology needs to be determined [135]. Although there are reviews [194,195] presenting thoroughly the recent clinical trials with OXT administration to humans, we are presenting here a limited number of them, mainly following intranasal administered OXT, which is the most commonly used route of administration in humans. Hence, there is markedly reduced hunger-driven and reward-driven food intake in men with obesity in contrast to normal-weight men [196], while male subjects with overweight and obesity led to bilateral hypoactivation in the ventral tegmental area (associated with hedonic regulation of human appetite) in response to viewing high-calorie food stimuli [162]. On the other hand, Ott et al. found no acute effects on total calorie intake in healthy, normal-weight men administered intranasal oxytocin and provided a buffet in the fasted state [163]. In a pilot study by Zhang et al. [164], four times daily intranasal OXT in Asian men and women with obesity for 8 weeks led to a BMI reduction. The magnitude of weight loss was also greater in subjects with higher degrees of obesity. In contrast, intravenous oxytocin infusion in 10 healthy men and women reduced satiety levels without affecting the volume of a liquid meal consumed [165]. A small improvement in insulin sensitivity assessed by oral minimal modelling was reported in normal-weight men administered acute intranasal oxytocin [166]. It is of importance that the same amount of intranasal OXT can either significantly reduce [167,168,196], or have no effect [163,169] on hunger-driven *ad libitum* caloric consumption.

Having presented the recent information on the human studies regarding the involvement of OXT in obesity/diabetes, we will present the basic research studies on this scientific issue. Starting with an *in vitro* model, in which the beneficial influence of oxytocin and its extended form to neonatal rat cardiomyocytes leading to their survival during metabolic stress was found [133]. Oxytocin also exerts a direct effect on macrophages derived from bone marrow by decreasing TNFα expression and secretion, which leads to the improvement of glucose metabolism [134].

Although the *in vivo* research for the OXT use in autism is mainly performed in rodents, notably genetically engineered mice, the equivalent research for OXT in obesity is performed in rodents, mainly rats, but also in other species, including non-human primates. The murine models include obese diabetic (ob/ob or db/db) mice [135,136], where reduction in lipid uptake, lipogenesis, was observed [135], and fat and glucose metabolism were partially improved [136] following oxytocin treatment. In leptin-resistant db/db mice, peripheral OXT injection suppresses food intake by activating vagal afferent neurons and, thereby, ameliorates obesity [137], while in the same animals, chronic OXT treatment also led to a reduction in visceral adipose tissue inflammation and plasma markers of systemic inflammation, which is believed to play a role in disease progression [138]. Studies in OXT KO mice have indicated that the genetic absence of OXT is associated with enhanced initial and sustained intake of sucrose solutions [139], and a strong link between OXT deficiency, late-onset obesity and decreased sympathetic tone [140]. In another study, OXTR−/− mice demonstrated that the oxytocin receptor plays essential roles in the regulation of energy homeostasis [141]. Other genetically engineered mice (Sim1 haploinsufficient) demonstrating hyperphagic obesity revealed the importance of OXT neurons in feeding regulation [142]. Diet-induced obese (DIO) mice have contributed vastly to the investigation concerning the link between obesity and oxytocin: Snider et al., demonstrated that selective activation of the OXT receptors pathway has benefits on lipid metabolism and weight loss [193]. The same authors showed concerns regarding the translation of their results to human obesity treatment since there studies were performed solely in male rodents. Others suggested that chronic hindbrain OXT treatment evokes sustained weight loss in DIO mice by reducing energy intake and increasing brown adipose fat thermogenesis at a dose that is not associated with evidence of visceral illness [143]. Labyb et al. demonstrated that chronic oxytocin treatment in C57BL/6JRj male mice on high fat diet improves the acute, but not the chronic leptin response, suggesting that this treatment could be used to improve the short-term satiety effect of leptin [144], while Snider et al. suggest the therapeutic benefits of OXT-related peptide analogs in both acute glycemic control and chronic lipid metabolism [193]. Lastly, Wu et al. [145] examined the effect of caffeine on PVN oxytocin neurons and regulation of energy balance in DIO mice, while Yuan J. et al. showed that C57BL6/J mice fed with high fat diet are in accordance with others, suggesting that chronic administration of oxytocin ameliorates obesity and metabolic dysfunctions [146]. Zhang, et al., showed that OXT and its analogs have multi-level effects in improving weight control, insulin sensitivity and insulin secretion, and bear potentials for being developed as therapeutic peptides for obesity and diabetes [164], not only in mice, but in humans as well. 

The work of Hayashi et al. also describes the effects of high-fat diet in other behaviors in mice, stating that OXT has the potential to improve various recognition memory processes via peripheral administration, but also has side effects that increase fear-related behavior in males [147], while others have found in OXT deficient mice that, in addition to the subsequent weight gain (adult-onset obesity), central oxytocin is involved in the thermoregulatory system as well. 

Zucker rats, have been used to study the metabolic effects of subchronic peripheral oxytocin administration [148], or the effects of reduced blood oxytocin [149] and the regulation of OXTRs in adipose and skeletal muscle [150]. High fat diet-fed rats, on the other hand, have been used to study the fat loss following chronic CNS oxytocin signaling [151] or chronic hindbrain administration [152], the mechanisms for the anti-obesity effects of oxytocin [153,154] and the combined effect of oxytocin with beta-3 receptor agonist to body weight and adiposity [155]. Other types of rats, such as the DHT-induced PCOS (polycystic ovary syndrome) model rat [156] and the Otsuka Long-Evans Tokushima Fatty (OLETF) rat have been used to study the effects of chronic OXT administration on body weight and food intake. These studies use female rats, following time-specific, post-weaning food restriction [157], and male rats in early-life food restriction [158] after the development of the model in 2009 [159].

Finally, species other than rodents, such as obese rhesus monkeys (fructose-fed), were studied following chronic oxytocin administration [160]. Intranasal oxytocin administration was also tested in prairie voles, where its administration reduced weight loss in diet-induced obese animals [161]. More insights from animal models on the potential of oxytocin as an anti-obesity strategy are listed in Blevins and Baskin publication [192].

*Ex vivo* studies in research of oxytocin in obesity/diabetes involvement do not exist.

### 3.4. In COVID-19

Lastly, all of these previously mentioned conditions, such as diabetes, high blood pressure, cardiovascular issues and obesity, are the ones that put the population at high-risk in coronavirus infection [197]. For this reason, oxytocin constitutes a biological target against the pandemic virus infection.

Early on the fight against SARS-CoV-2, scientists considered oxytocin as a potential therapeutic molecule, since it holds the ability to alleviate diseases such as cardiovascular entities, obesity and diabetes, which put covid patients at higher risk. Although there is a review on the possible effect of oxytocin to COVID-19 [198], the urgency for therapeutics in this issue produces more aspects on the potential use of oxytocin. Previous publications on oxytocin properties revealed: the interactions between nervous and immune system [199]; the fact that human vascular endothelial cells express oxytocin receptors [171]; the fact that when used in experimental LPS-induced acute lung injury in mice, oxytocin had a protective effect [170]; and the evidence that viral infections in humans (including influenza) attenuate oxy receptor expression, indicative of a key role of oxy system in human health [172]. Soumier and Sirigu [197], in a very brief and concise letter to the Elsevier editor, suggested oxytocin as a potential defensive biomolecule against COVID-19.

More publications and hypotheses came out following the previously mentioned comment about the use of oxy against the pandemic [200,201]. In 2020, a handful of publications examined oxy therapeutic use through different approaches, including the anti-inflammatory effects of oxytocin [202], presenting numerous animal studies on anti-inflammatory, antioxidative and restorative effects of oxytocin. Others [203] approached both the anti-inflammatory and pro-immune functions of oxy through transcriptomics, or as a natural dipeptidyl peptidase-4 (DPP4) protease inhibitor [204], which has been proposed for coronavirus treatment [205]. Some publications consider oxy as an antiviral agent [206,207] by inhibiting the SARS-CoV-2 main protease, while others [208] speculated that oxytocin analogues are inhibitors of the SARS-CoV-2 RNA-dependent RNA polymerase. 

More recent research [209] has shown the cardiovascular protection of oxytocin in the COVID-19 patients. This work also presents the possible complications or drawbacks from the use of this biomolecule to the treatment of the debilitating disease. 

From the previously mentioned research, is it evident that although there is a potential for the use of oxytocin into COVID-19 patients, animal studies are needed to explore oxytocin treatment on these patients. In an earlier study in mice [170] (Table 1) and in other studies presented in a review on the treatment of sepsis, among other diseases [210] OXT and related peptides might have a therapeutic role. Signs of multi-organ injury typical of sepsis occur in approximately 2–5% of COVID-19 patients 8–10 days post-infection, hence OXT potentially could alleviate this complication [211].

## 4. Conclusions

Oxytocin has been one of the most thoroughly investigated molecules. Its diverse functions have been known from the early years of its invention and/or have been identified over the years. Its use in dysfunctional situations both in humans and animals, especially for the confrontation of parturition adversities, is indispensable. Animal models have contributed the most to the use of this molecule in other degenerative disorders, related to nervous system or not. There are some points raised, especially the efficacy of oxytocin in aged populations. In particular, it has been indicated that in female rats, both the amount of plasma oxytocin and its receptor may decrease with age as a consequence of a diminished estrogen stimulation [212]. In male rats, there have been also reported a blunted oxytocin response to stress with age [213]. No investigations appear to have addressed the impact of advanced age on constitutive levels and responsiveness of oxytocin in humans [214]. However, the increasing incidence of osteoporosis with age, particularly in women, may be one indication of an attenuated influence of oxytocin in elderly people. 

Additionally, oxytocin interventions to ASD and other neurological disorders have been questioned. Ford and Young brought up the importance of context during behavioral OXT-assisted therapy [215], a fact that is also adopted by other researchers, such as Korisky et al. [216] and Althammer et al. [217]. They all claim that although OXT facilitates social learning, it does not directly cause prosocial behavior, and suggest that OXT treatment should be accompanied by behavioral therapy. The latter study also gives an explanation on the reason that OXT-treatment seems to be most efficient in infants, but not in adolescents. Hence, due to oxytocin’s distinct aspects in function, there is still plenty to explore for its uses in dysfunction.

Reviews and original papers are being published continuously [218] and among these publications, the present study aims to fill a gap by reviewing the biomolecules’ dynamics in the pursuit of alleviating degenerative diseases.

Conclusively we can hypothesize that oxytocin neural pathways interact closely with other pathways of the central nervous system responsible for processing motivationally relevant stimuli, and, hence, exert some of its social–behavioral effects. 

Natural oxytocin also acts on various endocrine pathways and its role includes molecular pathways that are definitely beyond parturition and milk-ejection. The affected molecular cell systems show potential long-term consequences and the manipulation of oxytocin system during childbirth may have a role in the early identification of women at risk for postpartum mood disorders or lactation difficulties [219]. The known neural connectivity and bursts of oxytocin during milk-ejection have a potential to identify differences in the genetic background of the animal and/or human, with implications in the diseases associated to agriculture with great financial consequences, e.g., in mastitis. 

Recent studies claim that oxytocin treatment in autism attenuates amygdala activity [220], improves thermo-sensory reactivity and maternal behavior in neonates lacking the autism-associated gene *Magel2* [221] or regulates the activities in ventromedial prefrontal cortex to alleviate the social deficits of autism [222]. The cellular OXT mechanisms involved in heart protection may include targeting of mitochondria, promoting glucose uptake, affecting the cardiac-renal axis and regulating the chronotropy and inotrophy of the heart [175]. Buemann and Uvnäs-Moberg present circulating oxytocin to enhance atrial natriuretic peptide production from the heart, PPAR-γ gene expression, PPAR-α activity and Ca2+ aiming to effect on cardiovascular risk profile [214]. Lastly, the OXT pathways that are possibly involved in obesity entail the inhibition of the activity of the food-related pathway, and the reward-related food motivation along with the decrease in food intake by controlling compensatory hedonic eating [223]. Kerem and Lawson propose a pathway for the effects of OXT on appetite regulation and food intake and metabolism which affects thermogenesis and brown adipogenesis [195].

The exact pathways of OXT effects in autism, cardiovascular diseases and obesity/diabetes are diverse. There is ongoing research on the clarification of the molecular mechanisms leading to the above-mentioned pathways. The *in vitro*, *in vivo* and *ex vivo* studies are assisting this research in order to strengthen the positive OXT effects on the remedy of these diseases and diminish the negative effects of this molecule.

Lastly, recent (last months of 2022) reviews present OXT as having the potential to become a key therapeutic agent for future treatment and prevention strategies concerning the main psychiatric disorders [224]. Others describe and discuss the data after having categorized the results presented in the articles according to certain subjects in sensory stimulation and OXY and their roles in social interaction and health promotion [225].

Additionally, since results from using oxytocin have been controversial, scientists are trying to extract information from the existing research in order to exclude all the negative effects of this biomolecule when used for the treatment of degenerative diseases. In this most recent review, authors are comparing different measuring methods of human OXT concentrations, and they point out the need to establish expert recommendations for study design and provide a rationale and a standardized set of recommendations for this reason when OXT is been implicated in numerous treatments [226].

It is obvious that oxytocin will continue to interest scientists as a possible treatment for various diseases, while they will try to minimize its negative effects. This review summarizes the current situation of the OXT use as a remedy for a specific group of diseases, and proposing that physiological, basic, well known, evolutionary documented actions of the element might be connected to the other different actions of this biomolecule. 

## Figures and Tables

**Table 1:** Studies (*in vitro*, *in vivo*, *ex vivo* and human) related to the use of oxytocin for the medication of degenerative diseases *.

Disease	*In Vitro* Studies	*In Vivo* Studies (Animal Models)	*Ex Vivo* Studies	Human Studies/Use
autism		OXTR deficient mice [39] OXT-deficient mice [98] CD38-deficient mice [100] OXT, OXTR Null Mice [101] OXTR Null Mice [102] Mice Heterozygous for the OXTR Gene [103] *Magel2^tm1.1Mus^*-deficient mice [104] *Magel1*-deficient mice [105] *Magel2*-deficient mice [106] *Magel2*-deficient mice [107] VPA rats and FRX mice [108] BALB/cByJ and C58/J mice [110,111] Oprm1−/− Mouse [112] HPC-1/Syntaxin1A Knockout Mice [113] *Shank3*-Deficient Rat [114]		Intranasal OXT in adolescent boys [87] Intranasal OXT treatment in ASD [88] Intranasal OXT in youth with ASD [89] Critical considerations for intranasal OXT in ASD [90] Systematic review and meta-analysis of adverse effects [91] Meta-analysis of OXT therapy in ASD [93] Systematic review for chronic OXT administration [94] Meta-analysis in intranasal OXT in ASD [95] Intranasal OXT in 3–17 years old people with ASD [96] Intranasal OXT in adults with ASD [109]
cardiovascular disease	macrophages [58] various cell types [116] myocytes, [117] rat newborn cardiomyocytes [118]	apoE−/− mice [119] Sprague-Dawley rats [120,121,122,123] Japanese white rabbits [124] WHHL rabbits [125] Porcine model [126]	rat heart [127,128,129,130] dog heart [131]	combined with obesity and/or diabetes
	H9c2 cells [132]			
obesity/diabetes	neonatal rat cardiomyocytes [133] mouse bone marrow cells [134]	**mice** obese diabetic ob/ob: [135] obese diabetic db/db: [136,137,138] OXT KO: [139,140], OXTRs KO: [141] Sim1 haploinsufficient mice [142] diet induced obesity: [143,144,145,146,147] **rats** zucker rats: [148,149,150] high-fat diet-rats: [151,152,153,154,155] DHT-induced PCOS model rats: [156] OLETF rats: [157,158,159] **other species** NHPs, [160] prairie voles, [161]		intranasal OXT, overweight and obese men: [162] intranasal OXT, ingestion and metabolic function in healthy men: [163] intranasal OXT in patients: [164] intravenous infusion of synthetic OXT analog to healthy men: [165] insulin sensitivity following intranasal OXT in healthy men: [166] intranasal OXT in fasted healthy men: [167,168] intranasal OXT in normal weight volunteers: [169]
COVID-19		mice [170]		OXTRs in human endothelial cells [171]
				effects of viral infection on OXTR [172]

* All the references from Table 1 are presented in Table 2, with the related result of oxytocin intervention and/or treatment of degenerative diseases.

**Table 2:** List of the studies (*in vitro*, *in vivo*, *ex vivo* and human) presented in Table 1 with related result of oxytocin intervention and/or treatment of degenerative diseases.

Autism	
*in vivo* studies	
Reference number	Effect of experimental intervention
[39]	developmental role for the OXT/OXTR system in shaping adult aggressive behavior
[98]	OXT is necessary for the normal development of social memory in mice; social memory has a neural basis distinct from other forms of memory
[100]	OXT release is influenced by CD38; in critically regulating maternal and social behaviors, and may be an element in neurodevelopmental disorders
[101]	OXT into the brain in a CD38-dependent manner may play an important role in the development of social behavior
[102]	*Oxtr*^−/−^ mouse is instrumental for the study of neurochemical and synaptic abnormalities underlying autistic-like disturbances and for testing new strategies of pharmacologic intervention
[103]	*Oxtr*^+/−^ mouse is a unique animal model for investigating how partial loss of the *Oxtr* gene impair social interactions, and for designing pharmacological rescue strategies
[104]	positive effects of subcutaneous administration of OXT in the mutant neonates (*Magel2^tm1.1Mus^*-deficient mice) restoring hippocampal alterations and social memory at adulthood
[105]	in human, *MAGED1* could play a role in autism or cause a neurodevelopmental condition that is reminiscent of the Prader–Willi syndrome
[106]	OXT plays a crucial role in setting social behaviors during a period just after birth
[107]	OXT supply might constitute a promising avenue for the treatment of feeding difficulties in Prader–Willi neonates
[108]	suggestion for a chronic deficient chloride regulation in the studied rodent models of autism and the importance of OXT-mediated GABAergic inhibition during the delivery process
[110]	BALB/cByJ and C58/J models as useful platforms for screening novel drugs for intervention in ASDs and for elucidating the mechanisms contributing to the prosocial effects of oxytocin
[111]	OXT can enhance sociability in mouse models with divergent genotypes and behavioral profiles
[112]	interaction between OXT and opioids in socially relevant brain areas and in the modulation of social behavior
[113]	STX1A plays an important role in social behavior through regulation of the OXTergic neural system
[114]	an oxytocinergic contribution in a genetically defined subtype of ASD (Phelan-McDermid syndrome) suggest an individualized therapeutic approach for this syndrome
human studies/use	
[87]	the first report documenting the safety of long-term nasal OXT therapy for children with ASD
[88]	daily administration of intranasal OXT at 0.4 IU/kg/dose in children and adolescents with ASD is safe and has therapeutic potential
[89]	a first evaluation of the efficacy for a course of OXT treatment for youth with ASD with results that did not suggest clinical efficacy
[90]	review stating that despite limitations in the field, remains significant potential for oxytocin to ameliorate aspects of the persistent and debilitating social impairments in individuals with ASD
[91]	systematic review supporting that intranasal OXT is well tolerated and safe for use in the ASD population
[93]	meta-analysis which demonstrated that OXT had a small and non-significant effect on core symptoms in ASD population
[94]	systematic review calling for more systematic and standardized research on chronic OXT administration in humans to supplement and expand what is currently known from preclinical work
[95]	OXT administration can be regarded as an effective treatment for some core aspects of ASDq; a meta-analysis
[96]	placebo-controlled trial of intranasal OXT therapy in children and adolescents with autism spectrum disorder showed no significant between-group differences
[109]	pilot study suggesting that there is therapeutic potential to daily administration of intranasal oxytocin in adults with ASD
**cardiovascular diseases**	
*in vitro* studies	
[58]	OXTR is an acute-phase protein and that its increased expression is regulated by NF-κB and functions to attenuate cellular inflammatory responses in macrophages
[116]	OXT attenuates vascular oxidative stress and inflammation
[117]	cGMP/protein kinase G mediates OXT-induced anti-hypertrophic response with the contribution of ANP and NO
[118]	supplementation of umbilical cord blood-derived mesenchymal stem cells (UCB-MSCs) with OXT can contribute to the cardiogenic potential for cardiac repair
[132]	OTR protected H9c2 cells against ischemia-reperfusion, especially if activated at the onset of ischemia or reperfusion
*in vivo* studies	
[119]	peripheral OT administration can inhibit atherosclerotic lesion development and adipose tissue inflammation
[120]	continuous OXT delivery reduces inflammation and apoptosis in infarcted and remote myocardium, thus improving function in the injured heart
[121]	OXT pretreatment inhibits the degranulation of cardiac mast cells induced by I/R injury and downregulates the expression of the inflammatory factors HMGB1 and NF-κB p65
[122]	activation of hypothalamic OXT neurons to elevate parasympathetic tone reduced cellular hypertrophy, levels of IL-1β, and fibrosis during trans-ascending aortic constriction (TAC)-induced heart failure in rats. Cardiac contractility parameters were significantly higher in TAC+OXT compared with TAC animals. Heart rate sensitivity, but not contractile sensitivity, to β-adrenergic stimulation was improved in TAC+OXT hearts
[123]	pre-autonomic OXT neurons can drive the increase in cardiac sympathetic nerve activity following myocardial infraction and peripheral administration of an OXT receptor blocker could be a plausible therapeutic strategy to improve outcomes for myocardial infraction patients
[124]	postinfarct treatment with OXT reduces myocardial infarct size and improves left ventricular function and remodeling by activating OXTRs and prosurvival signals and by exerting antifibrotic and angiogenic effects through activation of MMP-1, endothelial NO synthase, and vascular endothelial growth factor
[125]	a decrease in adipose tissue inflammation in the OXT-treated group compared to the vehicle control group, however these differences were not statistically significant
[126]	pretreatment endogenous OXT levels and timing of OXT administration post myocardial infraction seem to impact outcome in this porcine model
*ex vivo* studies	
[127]	attenuation of the infarct size in OXT-treated hearts, indicating a cardioprotective effect of oxytocin
[128]	oxytocin dose-dependently exerts cardiac postconditioning
[129]	i.c.v. infusion of exogenous OXT and centrally released endogenous oxytocin in response to stress could play a role in induction of a preconditioning effect in ischemic-reperfused rat heart via brain receptors
[130]	activation of cardiac OXT receptors by OXT released in response to stress may participate in cardioprotection and inhibition of myocardial ischemia/reperfusion injury
[131]	negative inotropic and chronotropic effects of oxytocin are mediated by cardiac oxytocin receptors and that intrinsic cardiac cholinergic neurons and NO are involved in these actions
**obesity/diabetes**	
*in vitro* studies	
[133]	OXT and OXT-GKR influence glucose uptake in neonatal rat cardiomyocytes and may thus play a role in the maintenance of cardiac function and cell survival during metabolic stress
[134]	OXT treatment decreases TNFα production both in vitro (in bone marrow-derived macrophages) and in vivo (in epididymal adipose tissue) in diet-induced obese mice.
*in vivo* studies	
[135]	imposes careful selection of the conditions in which OXT treatment should be beneficial for obesity and its comorbidities
[136]	chronic treatment with OXT partially improves glucose and fat metabolism and reverses abnormal cardiac structural remodeling, preventing cardiac dysfunction in db/db mice
[137]	peripheral OXT injection suppresses food intake by activating vagal afferent neurons and thereby ameliorates obesity in leptin-resistant db/db mice
[138]	chronic OXT treatment led to a reduction in visceral adipose tissue inflammation and plasma markers of systemic inflammation
[139]	central OXT pathways may normally participate in limiting initial intake of novel ingesta and may also participate in limiting intake of sweet, highly palatable familiar ingest
[140]	lack of hyperphagia evident in the OXT(−/−) mice may, in part, be attributed to the developmental compensation of other satiety factors such as cholecystokinin or bombesin-related peptides
[141]	OXTR plays essential roles in the regulation of energy homeostasis
[142]	reduced OXT neuropeptide is one mechanism mediating the hyperphagic obesity of Sim1(+/−) mice
[143]	selective activation of the OXTR pathway results in both acute and chronic metabolic benefits, whereas potential activation of vasopressin receptors by nonselective OXT analogs causes physiological stress that contributes to additional weight loss
[144]	chronic hindbrain OXT treatment evokes sustained weight loss in diet-induced obesity mice by reducing energy intake and increasing brown adipose tissue thermogenesis at a dose that is not associated with evidence of visceral illness
[145]	chronic OXT treatment improves the acute, but not the chronic leptin response, suggesting that this treatment could be used to improve the short-term satiety effect of leptin
[146]	caffeine inhibits A_1_Rs expressed on PVN oxytocin neurons to negatively regulate energy balance in diet-induced obesity mice
[147]	OXT induces inguinal white adipose tissue (iWAT) browning and stimulates thermogenesis in brown fat tissue, iWAT and skeletal muscle, through which OXT promotes thermogenesis and thus combats obesity and metabolic dysfunctions
[148]	ambivalent effects of OXT treatment with predominantly negative impact on skeletal muscle insulin pathway in lean animals
[149]	OXT system in the pathogenesis of obesity and suggests oxytocinase inhibition as a candidate approach in the therapy of obesity
[150]	OXTR is differentially regulated in adipose tissue of obese rats depending on fat depot localization
[151]	long-term 3V administration of OXT to rats can both prevent and treat diet-induced obesity
[152]	in diet-induced obesity rats, OXT action in the hindbrain evokes sustained weight loss by reducing energy intake and increasing brown fat tissue thermogenesis
[153]	OXT infusion failed to induce weight loss and fat oxidation in PPAR-alpha-deficient animals
[154]	OXT circumvents leptin resistance and induces weight-loss in diet-induced obesity animals through a mechanism involving activation of neurons in the NTS and AP, key hindbrain areas for processing satiety-related inputs
[155]	the effects of the combined treatment on energy intake, fat mass, adipocyte size and browning of epididymal white adipose tissue were not additive and appear to be driven, in part, by transient changes in energy intake in response to OXT
[156]	OXT could be a candidate drug for preventing the onset of obesity-related metabolic disorders in polycystic ovary syndrome patients
[157]	emphasis on the importance of sex-appropriate approaches in the investigation and treatment of the pathologies related to obesity and the metabolic syndrome
[158]	early interventions may effectively moderate obesity, even in the presence of a genetic tendency
[159]	importance of using different sex-appropriate approaches to increase the efficacy of therapeutic interventions in the treatment and prevention of chronic early-onset obesity
[160]	OXT reduces body weight in diet-induced obesity rhesus monkeys through decreased food intake as well as increased energy expenditure and lipolysis
[161]	OXT treatment reduces weight gain and body fat mass in diet-induced obesity prairie voles, in part, by reducing food intake
human studies/use	
[162]	OXT administration reduces activation in homeostatic and increases activation in cognitive control brain regions critically involved in regulating food intake and resolving affective conflict, respectively
[163]	OXT, beyond its role in social bonding, regulates nonhomeostatic, reward-related energy intake, hypothalamic-pituitary-adrenal axis activity, and the glucoregulatory response to food intake in humans
[164]	OXT and its analogs have multi-level effects in improving weight control, insulin sensitivity and insulin secretion, and bear potentials for being developed as therapeutic peptides for obesity and diabetes
[165]	infusion of OXT reduces satiety without affecting the volume of nutrient intake or gastric emptying in healthy subjects
[166]	OXT plays a significant role in the acute regulation of glucose metabolism in healthy humans and render the oxytocin system a potential target of antidiabetic treatment
[167]	specific role of OXT in the regulation of eating behavior in humans that might be of relevance for potential clinical applications
[168]	Intranasal OXT reduces caloric intake and has beneficial metabolic effects in men without concerning side effects. The efficacy and safety of sustained oxytocin administration in the treatment of obesity warrants investigation
[169]	No effects of intranasal OXT were seen in reward circuits or on ad libitum food intake
**COVID-19**	
*in vivo* studies	
[170]	OXT could reduce inflammatory responses of lipopolysaccharide-induced acute lung injury
human studies/use	
[171]	endothelial OXT receptors produce a calcium-dependent vasodilatory response via stimulation of the nitric oxide pathway and have a trophic action
[172]	unrelated viral pathologies have a common consequence: diminished levels of OXTR

## Data Availability

Not applicable.
